# The Specificity of Individuals with Depressive Tendencies in Processing Verbal Emotional Information: Evidence from ERP

**DOI:** 10.3390/bs16050666

**Published:** 2026-04-28

**Authors:** Dan He, Yutong Li

**Affiliations:** The School of Psychology, Liaoning Normal University, Dalian 116029, China; bxyzhd@163.com

**Keywords:** valence judgment, depressive tendency, verbal emotional information, event-related potential (ERP)

## Abstract

To explore the specificity of processing verbal emotional information by individuals with depressive tendencies, individuals with depressive tendencies and healthy individuals were asked to make valence judgments on words, and the changes in brain potential induced by words were recorded. The behavioral results show that compared with healthy individuals, individuals with depressive tendencies had a lower accuracy rate in judging the valence of words and showed a longer time to judge neutral words. The electroencephalogram (EEG) results revealed that when processing negative words, individuals with depressive tendencies induced smaller N400 amplitudes in the frontal region, front–central region, and central region than healthy individuals, and also induced a larger LPP with weakened energy in the alpha band. In the front–central region, central region and central–parietal region, individuals with depressive tendencies showed a greater induction of LPP amplitude when processing neutral words than healthy individuals. The above results suggest that individuals with depressive tendencies have specificity in processing verbal emotional information and slower and less accurate judgment of lexical valence, accompanied by specific changes in brain potential.

## 1. Introduction

With the increasing prevalence of depression, individuals with depressive tendencies have also attracted the attention of researchers. Although individuals with depressive tendencies do not meet the clinical diagnostic criteria for depression, they often exhibit persistent depression, helplessness and negative cognitive patterns (Cuijpers et al., 2021). A study suggests that individuals with depressive tendencies and those with depression may be on the same continuous spectrum (Angst & Merikangas, 1997), and that their cognitive function impairments are similar. The cognitive model of depression holds that individuals with depression have negative cognitive schemas and distort their perception of external information (Kovacs & Beck, 1978). Negative cognitive schemas lead individuals to have negative biases in attention, interpretation and memory, thereby exacerbating depressive symptoms (Disner et al., 2011). Research has found that patients with depression have difficulty in suppressing and releasing negative information, that is, impaired cognitive control (Joormann & Tanovic, 2015). Abnormal processing of negative information by working memory is the cognitive basis of negative bias in patients with depression (LeMoult & Gotlib, 2019).

Previous studies on the negative bias of depressed individuals mainly used faces, pictures, etc., as experimental materials (Duque & Vázquez, 2015; Klawohn et al., 2020). Apart from faces and pictures, words are also an important medium for conveying emotional information, but words are quite different from faces and pictures. First of all, words directly present semantic information, and the extraction of their emotional information needs to be carried out in combination with experience. Secondly, the emotional information presented by faces and pictures is explicit, while the emotional information conveyed by words is implicit (Peckham et al., 2010). Thirdly, pictures and faces can only convey the information they carry themselves, while words can not only convey literal information but also metaphorical information, and even information contrary to the literal meaning (irony). Therefore, it is worth studying how individuals with depressive tendencies perceive verbal emotional information. Studies have found that depressed individuals use more negative words (Lyons et al., 2018). Depressed individuals who overly focus on negative verbal information will increase their incidence of depressive symptoms (Joormann, 2010). Shane and Peterson (2007) found that when comparing neutral words with positive words, individuals with depressive tendencies paid more attention to neutral words, while healthy individuals paid more attention to positive words. Ellis et al. (2011) found that individuals with depressive tendencies spent more time fixating on negative words than healthy individuals. However, a study has found that there is no difference in the processing of neutral words between individuals with depressive tendencies and healthy individuals (Besche-Richard et al., 2002). It can be seen that the results of the above studies are not consistent. Whether the processing of verbal emotional information by individuals with depressive tendencies has particularity needs further verification.

Studies monitoring changes on an electroencephalogram (EEG) when individuals process verbal and emotional information using event-related potential (ERP) mainly focus on the amplitude changes in the N400 and LPP (Late Positive Potential, LPP) components. The N400 component reflects the cognitive process of the brain processing speech information. Changes in N400 amplitude are related to the difficulty of semantic integration. The greater the difficulty of semantic integration, the greater the amplitude of N400 (Kanske & Kotz, 2007). Kiang et al. (2017) found that when processing negative words, the N400 amplitude produced by depressed individuals was smaller than that of healthy individuals, indicating that it was easier for depressed individuals to process negative words. The amplitude changes in LPP usually reflect the sustained attention and evaluation processing of emotional stimuli (Fischler & Bradley, 2006; Herbert et al., 2006). A study of depressed female adolescents found that in the self-referential encoding task, compared with healthy subjects, depressed subjects produced a greater amplitude of LPP when processing negative words than positive words (Auerbach et al., 2015). In addition, a study found that brain waves in the alpha band were highly correlated with depressive symptoms (Aleksandra et al., 2023). Resting-state EEG data indicate that the alpha band power of depressed individuals is significantly weakened compared with that of healthy individuals. Further analysis revealed that the alpha power of the bilateral frontal and occipital lobes in patients with MDD was positively correlated with information processing rate, speech learning, working memory and attention maintenance (Xie et al., 2024). Enhanced alpha band power in the right frontal lobe can effectively improve the emotional, behavioral and cognitive problems of depressed individuals (Choi et al., 2010). Another study indicates that the power attenuation in the alpha band reflects an individual’s insufficient inhibitory control over task-irrelevant information (Jensen & Mazaheri, 2010), which may lead to increased difficulty for depressed individuals to disengage from negative information.

This study required individuals with depressive tendencies and healthy individuals to complete a lexical valence judgment task, and recorded the behavioral responses of lexical judgment and the changes in brain potential caused by words. The specificity of individuals with depressive tendencies in processing verbal emotional information was explored through the changes in N400, LPP amplitudes and alpha band energy. Based on the cognitive theory of depression, this study hypothesizes that individuals with depressive tendencies have developed negative cognitive schemas and have a tendency to prioritize processing negative information. In the late stage of processing, negative information gains sustained attention and is finely processed. Compared with healthy individuals, individuals with depressive tendencies produce smaller N400 amplitudes and larger LPP amplitudes when processing negative words. Moreover, the alpha band energy of individuals with depressive tendencies weakens when processing negative words. Furthermore, patients with depression tend to interpret pleasant faces as neutral faces and misjudge neutral faces as sad faces (Elliott et al., 2011). It is not clear whether individuals with depressive tendencies also negatively process neutral information in speech. The depression level of individuals with depressive tendencies is not as severe as that of patients with depression, and the emotional information conveyed through speech is also weaker than that in pictures (Frühholz et al., 2011). If it is observed that the processing mode of neutral information by individuals with depressive tendencies is also different from that of healthy individuals, this will indicate that individuals with depressive tendencies already possess cognitive biases for neutral information.

## 2. Materials and Methods

### 2.1. Participants

A two-factor mixed experimental design was adopted. The inter-subject variables were groups (depression tendency group, healthy control group), and the intra-subject variables were lexical valences (positive, neutral, and negative). The dependent variables are the reaction time and accuracy rate of valence judgment, as well as the changes in brainwave amplitude and power caused by valence judgment. The required sample size was estimated using G*Power 3.1.9.2 (α = 0.05, 1 − β = 0.90, effect size f = 0.25) (Faul et al., 2009), indicating a minimum of 28 participants. Ultimately, 40 college students were recruited.

The subjects were screened from 3500 undergraduate students using the Chinese Version of the Beck Depression Inventory-II (BDI-II-C). The formal experiment was conducted within one week after BDI-II-C screening. Before the experiment, all the subjects filled out the Center for Epidemiologic Studies Depression Scale (CES-D). Subjects with a BDI-II-C score of ≤13 points and a CES-D score of <20 points were screened out and included in the healthy control group, while subjects with a BDI-II-C score of >13 points and a CES-D score of ≥20 points were included in the depression tendency group.

In addition, according to the Structured Clinical Interview manual of the DSM-5 (SCID-5), structured interviews were conducted by two professionally trained graduate students. Exclusion criteria were those whose symptoms were attributed to recent major stressful or traumatic events (such as the death of a loved one); individuals with bipolar disorder, major depressive disorder and organic mental disorders of the brain; individuals who take medication regularly; and individuals with a history of neurological diseases. Finally, 22 subjects (age: 19.82 ± 1.37 years, 5 males and 17 females) in the depressive tendency group and 21 subjects (age: 20.43 ± 1.08 years, 6 males and 15 females) in the healthy control group were screened out, totaling 43 subjects. All the subjects were right-handed, with normal vision or corrected vision. The relevant information of the subjects is shown in Table 1. This study was approved by the university Ethics Committee with the approval number LL2024173. Before the experiment, the participants signed the informed consent form and received a certain amount of monetary remuneration after the experiment.

### 2.2. Stimuli and Procedure

Strokes are the smallest components that make up Chinese characters. For instance, 王 (wang) is composed of three “horizontal” (一) and one “vertical” (|) stroke. Strokes form Chinese characters, and Chinese characters form words. Many words are formed by two Chinese characters, such as “工人” (worker), “教师” (teacher), etc. We chose 80 positive, 80 negative and 80 neutral two-character words. There were no significant differences among the three types of words in terms of the number of strokes in the first character, the number of strokes in the last character, the number of strokes in the entire word, the frequency of the first character, the frequency of the last character, and the frequency of the entire word (*p*s > 0.05). Word frequency data were taken from the SUBTLEX-CH corpus (Cai & Brysbaert, 2010). Fifteen college students who did not participate in the formal experiment were recruited to rate the emotional valence, arousal and concreteness of the vocabulary on a 7-point scale (1 represents very unpleasant, very calm, or very abstract; 7 represents very pleasant, very excited, or very concrete). The valence score was analyzed. The main effect of lexical valence was significant, *F* (2, 28) = 165.66, *p* < 0.001, η_p_^2^ = 0.92. Positive words had the highest score, followed by neutral words, and negative words had the lowest score, with *p*s < 0.001. The arousal score was analyzed, and the main effect of lexical valence was significant, *F* (2, 28) = 21.05, *p* < 0.001, η_p_^2^ = 0.60. The score of emotional words was significantly higher than that of neutral words, with *p*s < 0.001, while there was no significant difference in the arousal score between positive words and negative words, *p* = 0.161. The analysis of the specificity score showed that the main effect of lexical valence was not significant, with *F* (2, 28) = 1.19 and *p* = 0.320. The data are shown in Table 2.

**Figure 1:**
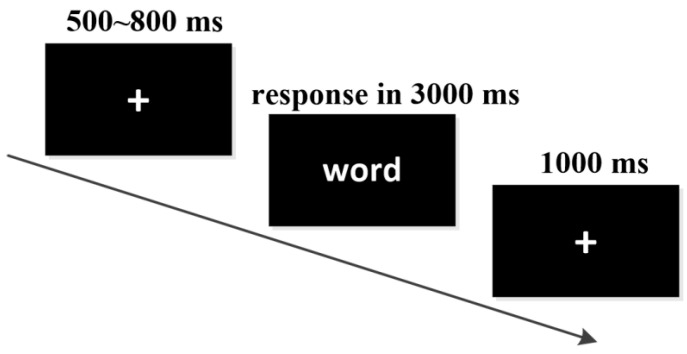
The procedure of a trial in the experiment.

The experimental program was written using E-Prime 2.0 software. The stimulus was presented in 32-point Song typeface, and the distance between the subject and the screen was 60 cm. The experimental process is shown in Figure 1.

First, a fixation point is presented for 500 to 800 ms. Then, an emotional word is presented for 3000 ms. The subjects need to make a valence judgment on the words. If the word is positive, press the f key with the left index finger. If the word is negative, press the j key with the right index finger. If the word is neutral, press the space bar with the thumbs of both hands (the f/j keys correspond to the valence, and balance among the subjects). If the subject does not press the button within 3000 ms, the stimulus disappears. Then, a 1000 ms blank screen is displayed and the next test is conducted. Before the official trial begins, six practice trials are presented first to familiarize the participants with the experimental process. The formal experiment consists of 3 blocks, each containing 80 trials, with a 2 min break between the blocks. The entire experiment lasted approximately 20 min.

### 2.3. EEG Recording

EEG data were recorded using a 64-channel Brain Products system. Electrode FPz served as the ground, and FCz was used as the online reference. Vertical electrooculogram activity was recorded using an electrode placed below the right eye to monitor blinks and vertical eye movements. Electrode impedances were maintained below 5 kΩ throughout the recording, and EEG signals were digitized at a sampling rate of 1000 Hz.

Offline preprocessing was performed using a BrainVision Analyzer 2.0 (Brain Products GmbH, Gilching, Germany). The data were re-referenced to the average of the left and right mastoids and filtered using a 0.1–30 Hz bandpass filter (24 dB/octave). Ocular artifacts were corrected with independent component analysis. The corrected EEG was segmented into epochs from −200 ms to 800 ms relative to stimulus onset, with the −200 to 0 ms interval used for baseline correction. Epochs containing artifacts exceeding ±100 μV in peak-to-peak amplitude were rejected.

### 2.4. Data Analysis

Time-domain analysis: The segmented data are from 200 ms before the target word appears to 800 ms after its appearance, with baseline correction from 0 to 200 ms before stimulation. Time-domain analysis focuses on the variation in brainwave amplitudes over time, with the core objective of extracting the components precisely locked in time with the stimulus. The incubation period of these components is usually within tens to hundreds of milliseconds, and they have extremely high requirements for time accuracy. The baseline window 200 ms before stimulation can cover the interference of low-frequency noise (such as breathing and blinking artifacts). Longer baselines (such as 500 ms) may introduce residual activities from early cognitive processing (such as CNV waves), contaminating baseline stability.

The amplitude of the effective trials under each condition is superimposed and averaged. Based on the relevant literature (Wang et al., 2019; Zhang et al., 2019), N400 (300–450 ms) and LPP (460–660 ms) were selected for analysis.

**Figure 2:**
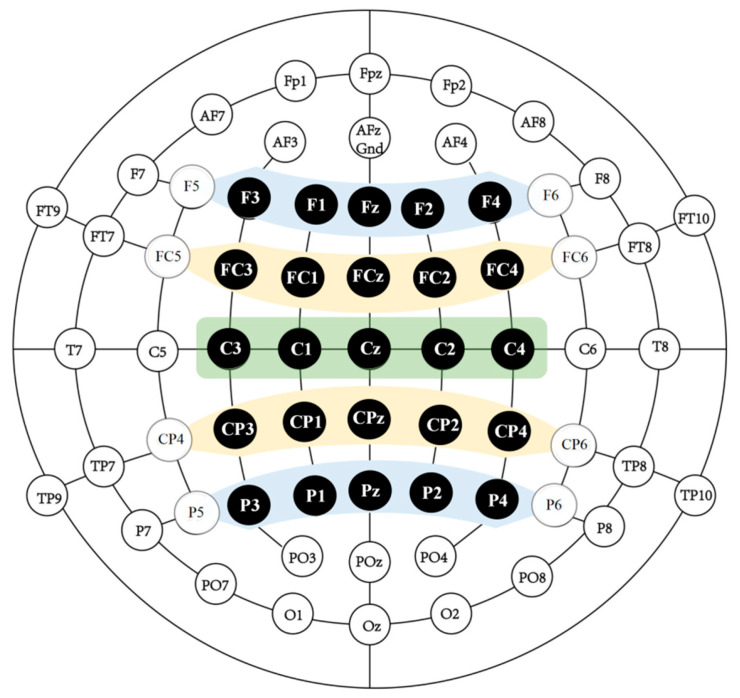
Topographical clusters and electrode positions.

The area of interest are divided into the prefrontal area (Fz, F1, F2, F3, F4), the frontal–central area (FCz, FC1, FC2, FC3, FC4), the central area (Cz, C1, C2, C3, C4), the central–parietal area (CPz, CP1, CP2, CP3, CP4), and the parietal area (Pz, P1, P2, P3, P4), as shown in Figure 2.

The data of the electrodes recorded in these areas are averaged and used for data analysis. Analysis of variance was conducted on the average amplitudes of N400 and LPP. The analysis factors included groups (depressive tendency group, healthy control group), valence of emotional words (positive, negative, neutral), and electrode regions (frontal region, frontal–central region, central region, central–parietal region, parietal region).

Time-frequency analysis: Re-extract the preprocessed EEG data, with the segmented data ranging from 500 ms before the presentation of the target word to 800 ms after its appearance. Unlike time-domain analysis, time-frequency analysis needs to simultaneously capture the frequency components of electroencephalogram (EEG) signals (such as α waves and β waves) and their changes over time. In time-frequency analysis, long windows (such as 500 ms) can enhance frequency resolution (for instance, precisely distinguishing α waves of 8–12 Hz).

Using the letswave7 toolkit, the EEG data was transformed into time-frequency domain data by wavelet variation. The wavelet function was complex Morlet, the bandwidth and center frequency were set to 1 and 1.5, and the frequency band range was 1–30 Hz. Then, the time-frequency representations of each test under certain conditions were summarized and the average taken to extract the event-related spectral oscillations on each frequency band. To avoid wavelet edge artifacts, the period from 500 ms before the stimulus presentation to the stimulus presentation is taken as the baseline. The baseline only includes pre-stimulation. The baseline correction is carried out using the formula ER% = (Xi − Mb)/Mb × 100%. Here, Xi represents the signal energy at a specific time and in a specific frequency band, and Mb represents the average energy of the baseline at a specific frequency band. Analyze the energy changes in the alpha wave (8–13 Hz) within the time windows of the N400 component (300–450 ms) and the LPP component (460–660 ms). The time windows for time-frequency analysis are selected as 300–450 ms and 460–660 ms, which respectively cover the core periods of N400 and LPP. The alpha power variation may occur earlier than the N400 peak, suggesting that the alpha oscillation is an early sign of semantic conflict detection, while N400 reflects the subsequent conflict resolution process. The continuous positive wave of LPP may be accompanied by the gradual recovery of alpha power, reflecting the attention to the redistribution of resources after emotional processing. Using the same electrode region division as in the ERP analysis, the average values of the data within the corresponding time-frequency range were analyzed by three-factor repeated-measures ANOVA for groups (depressive tendency group, healthy control group), word value (positive, negative, neutral), and electrode regions (frontal region, front–central region, central region, central–parietal region, parietal region).

The exported e-prime data and the data after electroencephalogram preprocessing were statistically analyzed using SPSS 27.0. The Greenhouse–Geisser method was used for correction, and the Bonferroni method was used for post hoc multiple comparisons. In addition, this study focused on group and word value. The following results will only present the significant results related to these two independent variables.

## 3. Results

### 3.1. Behavioral Data

Due to the artifacts in the electroencephalogram (EEG) data, one subject each was excluded from both the depression tendency group and the healthy control group. The reaction time data that are greater than or less than the mean plus or minus 2.5 standard deviations were deleted. Here, “*SD*” refers to the *SD* of each subject for each condition. We conducted data analysis on the trials that responded correctly. The statistical results are described in Table 3. Compared with the healthy group, the depression group misjudged positive words as neutral words by 4.15% more, neutral words as negative words by 6.63% more, and negative words as neutral words by 4.35% more.

Repeated-measures analysis of variance (ANOVA) of 2 (groups: healthy control group, depression tendency group) × 3 (valence of emotion words: positive, neutral, negative) was conducted on the results for reaction time and accuracy rate, respectively.

Repeated-measures analysis of variance was conducted on the accuracy rate. The results showed that the group main effect was significant, *F* (1, 39) = 8.01, *p* = 0.007, η_p_^2^ = 0.17. The accuracy rate of the depression tendency group was lower than that of the healthy control group. The main effect of word valence was significant, *F* (2, 78) = 27.48, *p* < 0.001, η_p_^2^ = 0.41. The accuracy rate of negative words is lower than that of neutral words, *p* < 0.001; the accuracy rate of negative words is higher than that of positive words, *p* = 0.026; and the accuracy rate of positive words is lower than that of neutral words, *p* < 0.001. The interaction between groups and word valence was not significant, *F* (2, 78) = 0.55, *p* = 0.580.

Repeated-measures analysis of variance was conducted on reaction time. The results showed that the main effect of the group was not significant, *F* (1, 39) = 1.89, *p* = 0.177, and the main effect of word valence was not significant, *F* (2, 78) = 0.15, *p* = 0.860. However, the interaction between groups and word valence was significant, *F* (2, 78) = 3.44, *p* = 0.037, η_p_^2^ = 0.08. Simple effect analysis revealed that there was no significant difference in reaction time between the groups on positive words (*p* = 0.128) and negative words (*p* = 0.826). Regarding neutral words, the reaction time of the depression tendency group was longer than that of the healthy control group, *p* = 0.055.

In conclusion, the accuracy rate of the depression tendency group was lower than that of the healthy control group. In terms of the reaction time to neutral words, the depression tendency group was longer than the healthy control group, and the difference reached a marginally significant level.

### 3.2. Time-Domain Results of EEG (Electroencephalogram)

**Figure 3:**
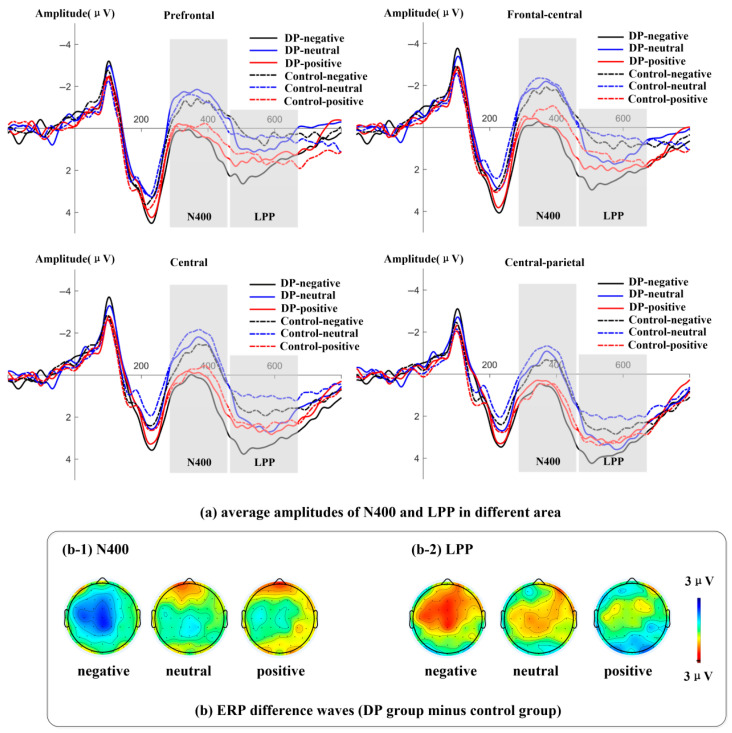
The amplitude changes and differential wave topography of the N400 component and LPP component caused by different valence terms in the two groups of subjects.

Table 4 describes the average amplitudes of N400 and LPP components induced by the two groups of subjects. Figure 3 depicts the average amplitude changes in the N400 component and the LPP component caused by processing different valence terms in the two groups of subjects.

From Table 4, it can be seen that some of the amplitudes of N400 are positive. Combined with Figure 3, it can also be seen that the peak value of the positive wave before the N400 wave is too high. After that, the N400 wave rises to a certain extent and reaches the peak, and the value at the peak remains positive. Then it begins to decline, forming a wave with a negative direction.

(1) N400 (300–450 ms)

**Figure 4:**
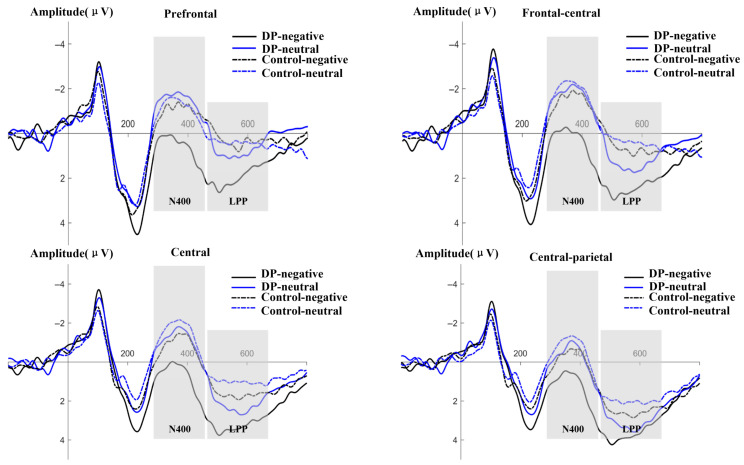
The amplitude changes and differential wave topography of the N400 component and LPP component caused by negative and neutral words in the two groups of subjects.

Analysis of variance revealed that the main effect of valence was significant, *F* (2, 78) = 11.66, *p* < 0.001, η_p_^2^ = 0.23. Both negative and positive words induced smaller N400 amplitudes than neutral words, with *p*s < 0.001. However, there was no significant difference between negative and positive words, *p* = 0.442. The main effect of the group was not significant, *F* (1, 39) = 2.54, *p* = 0.119. The third-order interactions among emotion word valence, groups and electrode regions were significant, with *F* (8, 312) = 3.29, *p* = 0.021, η_p_^2^ = 0.08. A simple effect analysis shows (see Figure 4) that for negative words, the N400 amplitude induced by the groups with depressive tendencies in the frontal area (a), the frontal–central area (b), and the central area (c) is smaller than that of the healthy control group (as shown in Figure 3). Frontal area: ∆a = 3.53, *p* = 0.047, 95% CI [0.05, 7.00]. Frontal–central region: ∆b = 3.36, *p* = 0.041, 95% CI [0.14, 6.57]. Central region: ∆c = 3.07, *p* = 0.044, 95% CI [0.09, 6.05]. For neutral words and positive words, no differences between groups were found in any electrode region, with *p*s > 0.162.

(2) LPP (460–660 ms)

Analysis of variance revealed that the main effects of valence (*F* (2, 78) = 1.79, *p* = 0.180) and group (*F* (1, 39) = 4.00, *p* = 0.053) were not significant. However, the third-order interactions of valence, group and electrode region were significant, *F* (8, 312) = 2.04, *p* = 0.041, η_p_^2^ = 0.05. A simple effect analysis showed (see Figure 4) that for negative words, the LPP amplitude induced in the depressive tendency group was greater than that in the healthy control group in the prefrontal area (a), the front–central area (b), and the central area (c) (see Figure 3). Prefrontal area: ∆a = 5.33, *p* = 0.035, 95% CI [0.38, 10.27]. Frontal–central region: ∆b = 4.78, *p* = 0.029, 95% CI [0.51, 9.06]. Central region: ∆c = 4.04, *p* = 0.037, 95% CI [0.26, 7.82]. For neutral words, in the frontal central region (b), central region (c), and central–parietal region (d), the groups with depressive tendencies all showed greater LPP amplitude induced than the healthy control group (as shown in Figure 3). Central region: ∆b = 4.48, *p* = 0.031, 95% CI [0.43, 8.53]. Central region: ∆c = 3.93, *p* = 0.029, 95%CI [0.43, 7.42]. Central–parietal region: ∆d = 3.54, *p* = 0.026, 95% CI [0.44, 6.64]. For positive words, no inter-group differences were found in any electrode region, with *p*s > 0.075.

In conclusion, the two groups of subjects produced different electrophysiological responses to the processing of negative words. Specifically, in the prefrontal area, frontal–central area and central area depression tendency groups, the amplitude of the N400 wave was smaller and the amplitude of the LPP wave was larger than that of the healthy control group. In addition, there were also differences in the late stage of neutral word processing between the two groups of subjects. That is, the LPP amplitude induced by the depression tendency groups in the frontal–central area, central area and central–parietal area was greater than that of the healthy control group.

### 3.3. Time-Frequency Domain Results of EEG

(1) alpha (300–450 ms)

Repeated-measures analysis of variance was conducted on the alpha wave power values (see Table 5). The valence main effect was not significant, with *F* (2, 78) = 0.60 and *p* = 0.554. The main effect of the group was not significant, *F* (1, 39) = 1.34, *p* = 0.254. In addition, the valence of emotion words and the second-order and third-order interactions involved in groups were not significant, with *F*s < 0.30 and *p*s > 0.783.

(2) alpha (460–660 ms)

**Figure 5:**
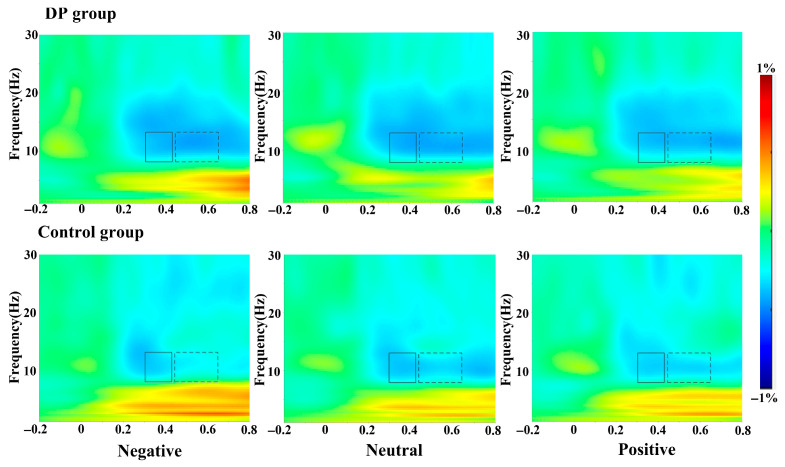
The results of event-related spectral oscillations of the α band in the 300–450 ms (solid box) and 460–660 ms (dashed box) of the two groups of subjects under different valence terms on the Fz electrode.

Repeated-measures analysis of variance was conducted on the alpha wave power values (see Table 5). The valence main effect was not significant, *F* (2, 78) = 2.36, *p* = 0.101. The group main effect was not significant, *F* (1, 39) = 2.95, *p* = 0.094. The third-order interactions of valence, group and electrode region were significant, with *F* (8, 312) = 2.63, *p* = 0.045, η_p_^2^ = 0.06. A simple effect analysis shows that for negative words, the alpha wave energy of the healthy control group is significantly higher than that of the depression tendency group in the frontal area (a), the frontal–central area (b), and the central area (c) (see Figure 5). Frontal area: ∆a = 1.74, *p* = 0.024, 95% CI [0.02, 032]. Frontal–central region: ∆b = 1.64, *p* = 0.036, 95% CI [0.01, 032]. Central region: ∆c = 1.16, *p* = 0.042, 95% CI [0.01, 032]. No energy differences between groups were observed in the electrode regions for positive and neutral words, with *F*s < 3.21 and *p*s > 0.081.

In conclusion, for negative words, compared with the healthy control group, the depression tendency group showed a weakened alpha wave energy in the frontal area, the frontal–central area and the central area.

## 4. Discussion

By using the word valence judgment task combined with ERP recording technology, the specificity and neural basis of the processing of Chinese emotional information by individuals with depressive tendencies were explored. The behavioral results show that compared with healthy individuals, individuals with depressive tendencies have a lower accuracy rate in vocabulary judgment and very likely need more time to judge neutral words. The electroencephalogram (EEG) results showed that when processing negative words, the depression tendency group induced smaller-amplitude N400 and larger-amplitude LPP in the frontal region, front–central region and central region compared with the healthy control group, and the increased LPP was accompanied by weakened energy in the alpha band. In addition, compared with the healthy group, the depression-prone group induced greater-amplitude LPP in the frontal–central region, central region and central–parietal region when processing neutral words. This study demonstrated the specificity of the response of individuals with depressive tendencies to Chinese emotional information at the EEG level. The main findings of this study will be discussed in detail below.

Firstly, the accuracy rate of word valence judgment in the depressive tendency group was lower than that in the healthy control group, indicating that individuals with depressive tendencies have significant deviations in the processing of verbal emotional information. It can be inferred that individuals with depressive tendencies have developed negative cognitive schemas, which affect the processing of verbal emotional information. Studies have found that patients with depression tend to process neutral faces into sad faces and happy faces into neutral faces (Bourke et al., 2010; Elliott et al., 2011). Individuals with depressive tendencies may already exhibit the same behavior as those with depression, tending to mistake positive words for neutral ones and neutral words for negative ones, resulting in a relatively low accuracy rate. In addition, the fact that the depression tendency group very likely needed more time to judge neutral words also confirms this view. Individuals with a depression tendency are inaccurate in judging neutral information and tend to mistake neutral information for negative information. However, negative cognitive schemas make the integration of negative semantic information easier and do not make the integration of positive semantic information more difficult. One reason is the “selective capture” of cognitive resources by negative schemas, which prioritizes the screening of information related to depression. When individuals are exposed to semantic information, negative schemas actively activate memories, emotions and thinking patterns related to depression, concentrating cognitive resources highly on negative content. The second reason is that negative schemas form a one-way reinforcement cycle of negative information through “attentional bias” and “interpretive bias”. However, positive information lacks similar processing paths and can be difficult to use to form stable representations in the cognitive system. The third reason is the low matching degree between positive information and negative schemas. The content of positive information has an essential conflict with the structure of negative schemas and is difficult to directly integrate by schemas.

Our research found that the depression-prone group showed significant neural response specificity in processing negative words, that is, compared with the healthy group, the depression-prone group induced smaller N400 amplitudes and larger LPP amplitudes in the frontal region, frontal–central region and central region. Research has found that N400 is associated with semantic integration. The smaller the amplitude of N400, the easier semantic integration is (Kanske & Kotz, 2007). We found that the N400 amplitude induced from the frontal area to the central area in the group with depressive tendencies was smaller than that in the healthy group, indicating that individuals with depressive tendencies have relatively easier semantic integration of negative words. From the perspective of Beck’s cognitive theory of depression, individuals with depressive tendencies have formed negative cognitive schemas, which make negative words match their internal cognitive schemas, thereby making the processing of negative words easier. This is consistent with the research results of Kiang et al. (2017). They also found that compared with healthy individuals, depressed individuals had smaller N400 fluctuations induced by processing negative words. This indicates that in the memory systems of individuals with depression, the memory of negative information or negative characteristics is more profound. Individuals with a tendency towards depression are more likely to process negative words, and the N400 amplitude triggered by negative words is smaller. In addition, studies have found that an increase in LPP amplitude indicates that the emotional stimulus has received sustained attention and that the emotional stimulus has undergone further evaluation and processing (Fischler & Bradley, 2006; Herbert et al., 2006). This study found that compared with the healthy group, the LPP amplitude induced in the frontal region to the central region was greater in the depressive tendency group when processing negative words, indicating that individuals with depressive tendencies conducted continuous attention and fine processing of negative words (Schupp et al., 2000). This is consistent with the research results of Auerbach et al. (2015). In addition, some studies suggest that rumination can lead to an increase in LPP amplitude, and rumination is closely related to depression (Lewis et al., 2015). That is to say, the continuous processing of negative words is related to rumination and aggravates depressive symptoms. The electroencephalogram (EEG) results suggest that individuals with depressive tendencies exhibit dual characteristics of semantic integration and facilitation in the early stage and excessive processing in the late stage when processing negative words. The results of this study indicate that negative cognitive schema were formed for individuals with depressive tendencies. They continuously invested attention resources to maintain the processing of negative information and showed difficulty in disengaging from negative verbal information. In addition, compared with the healthy control group, there was no difference in the processing of neutral words in the depressive mood group at the N400 window, but there was a significant difference at the LPP window, with a larger LPP amplitude. It is very likely that the negative bias towards neutral words was not manifested in the early stage of processing, and the negative bias began to take effect in the late stage of processing. This re-evaluation of the negative tendency towards neutral words consumes more energy. Of course, its specific mechanism remains to be further explored.

In depression research, alpha waves can be used as pathological markers, and their dynamic changes can reflect the effect of therapeutic intervention (Aleksandra et al., 2023). In this study, the processing of negative words in the depressive tendency group led to a decrease in late alpha wave power compared to the healthy group, which was consistent with the result of weakened alpha band power in patients with depression (Xie et al., 2024). Weakened alpha power in the bilateral frontal and occipital lobes leads to decreased functions such as information processing rate, speech learning, and attention retention in depressed individuals (Xie et al., 2024). An increase in the power of the alpha band is associated with functional inhibition in task-independent brain regions, while a decrease in power indicates the lifting of inhibition and enhanced activation or information processing in task-related brain regions (Jensen & Mazaheri, 2010). Of course, there is also a possibility that an increase in the power of the alpha band will occur if the brain cortex is highly alert. A decline in brain alertness leads to a decline in the power of the alpha band. The decline in the late alpha wave power of negative word processing in the depressive tendency group indicates that the inhibitory control function of negative information in individuals with depressive tendencies is impaired. The release of this inhibition may lead to the continuous activation of negative information in working memory, thereby exacerbating attention disengagement difficulty (Koster et al., 2011), forming a vicious cycle.

In addition, the electroencephalogram (EEG) results and behavioral results of this study consistently revealed that individuals with depressive tendencies exhibited abnormal patterns in processing neutral words. In terms of behavior, the group with a tendency towards depression took longer to judge neutral words than the healthy group. In terms of electroencephalogram (EEG) results, the depressive tendency group induced a greater amplitude of LPP from the frontal area to the central–parietal area than the control group. That is to say, individuals with depressive tendencies find it difficult to quickly label neutral information as emotionless, and it takes longer and more cognitive resources to process neutral words. In addition, it is also possible that individuals with depressive tendencies attribute emotional meanings to neutral words, leading to an emotional assessment process in the brain in the later stage. This indicates that individuals with depressive tendencies may already have a cognitive tendency to “negate” neutral information.

This study has the following limitations and future research needs to be improved. First, standardization of experimental materials. The valence control among different words is effective, but there are also differences in the arousal degree among words, although the difference in arousal degree among emotion words is not significant. Future research can further control the differences in arousal and improve internal validity. Second, there is a bias in the gender ratio of the subjects. In this study, the subjects were selected from normal universities with a relatively high proportion of female students, and the composition of the subjects was mostly female. Although the proportion of subjects is consistent with the gender ratio of the population, gender ratio bias will affect the extrapolation validity. Future research should further control the gender ratio to enhance the validity of extrapolation. Thirdly, during the experimental design stage, we constantly tried different key schemes. The selection of the F/J button became a habit. We also wanted to use the space bar to correspond to emotion words, but the effect was very poor and the error rate increased significantly. Finally, fixing the neutral word to correspond with the space bar led to habitual reactions, and the resulting errors affected the power of the experiment. It is necessary for future research to explore more effective key-pressing schemes.

## 5. Conclusions

Individuals with depressive tendencies have specific manifestations when processing the emotional information expressed by Chinese characters. At the behavioral level, it is manifested as slow judgment speed and low accuracy. At the brain foundation level, individuals with depressive tendencies show a neural activity pattern of reduced N400 amplitude and increased LPP amplitude when processing negative words, accompanied by weakened alpha energy. However, for neutral information, they exhibit an abnormal processing pattern similar to emotional information, accompanied by increased LPP amplitude. In conclusion, individuals with depressive tendencies have negative biases and cognitive biases towards neutral information, which may be related to weakened inhibitory control and abnormal resource allocation. This indicates that individuals with depressive tendencies have formed negative cognitive schemas, which are supported by electroencephalogram (EEG) data.

## Figures and Tables

**Table 1:** Demographic information of the subjects (*M* ± *SD*).

Item	Healthy Group	Depression Tendency Group	*p* Value
Gender (M/F)	6/15	5/17	0.661 ^a^
Age	20.43 ± 1.08	19.82 ± 1.37	0.113 ^b^
BDI-II	3.33 ± 3.62	18.18 ± 5.54	<0.001 ^b^
CES-D	7.57 ± 5.09	25.45 ± 6.24	<0.001 ^b^

Note: a—Chi-square test; b—independent sample *t*-test; M—male; F—female.

**Table 2:** Descriptive statistics of vocabulary (*M* ± *SD*).

Attribute	Valence		
	Positive Word	Neutral Word	Negative Word
The number of strokes in the first character	9.19 ± 3.14	8.39 ± 3.26	9.05 ± 3.21
The number of strokes in the last character	8.56 ± 3.14	9.00 ± 3.25	8.80 ± 2.99
The number of strokes in the entire word	17.75 ± 4.34	17.39 ± 4.15	17.85 ± 4.27
The frequency of the first character (logarithm)	4.11 ± 0.76	4.11 ± 0.56	3.92 ± 0.71
The frequency of the last character (logarithm)	4.05 ± 0.76	4.09 ± 0.74	3.87 ± 0.70
The frequency of the entire word (logarithm)	2.36 ± 0.78	2.61 ± 0.70	2.51 ± 0.64
Valence score	5.90 ± 0.15	4.15 ± 0.39	2.32 ± 0.13
Arousal score	4.38 ± 0.37	2.43 ± 0.29	4.05 ± 0.36
Concreteness score	3.51 ± 0.23	3.77 ± 0.16	3.51 ± 0.23

Note: Strokes are the smallest components that make up Chinese characters. For instance, 王 (wang) is composed of three “horizontal” (一) and one “vertical” (|).

**Table 3:** Reaction time and accuracy rate of word value judgment (*M* ± *SD*).

	Depression Tendency Group			Healthy Control Group		
	Negative	Neutral	Positive	Negative	Neutral	Positive
Accuracy (%)	85.71 ± 6.67	91.31 ± 9.94	83.10 ± 12.09	90.06 ± 3.02	97.94 ± 1.53	87.25 ± 5.39
Reaction time (ms)	838 ± 172	875 ± 205	838 ± 172	828 ± 114	797 ± 107	796 ± 99

**Table 4:** Average amplitude of N400 and LPP components in different regions [*M* (*SD*)].

	Area	Depression-Prone Group			Control Group		
		Positive	Neutral	Negative	Positive	Neutral	Negative
N400	prefrontal	0.96 (4.69)	−0.81 (4.73)	1.21 (4.81)	−1.40 (6.78)	−2.59 (5.60)	−2.32 (6.12)
	frontal–central	0.53 (4.79)	−1.13 (4.66)	0.80 (5.04)	−1.74 (5.68)	−3.09 (4.60)	−2.55 (5.13)
	central	0.59 (4.40)	−0.82 (4.49)	1.01 (4.74)	−1.23 (5.25)	−8.72 (4.03)	−2.07 (4.70)
	central–parietal	1.11 (3.91)	−0.01 (4.16)	1.45 (4.36)	−0.25 (4.79)	−1.71 (3.54)	−1.10 (4.17)
	parietal	1.01 (3.37)	0.12 (3.53)	1.29 (3.80)	0.17 (4.30)	−0.98 (2.99)	−0.32 (3.66)
LPP	prefrontal	2.10 (4.27)	1.50 (4.02)	2.56 (4.34)	1.24 (3.67)	0.21 (3.71)	0.76 (3.17)
	frontal–central	2.42 (4.48)	1.93 (4.05)	2.86 (4.98)	1.36 (3.01)	0.11 (3.47)	1.02 (2.79)
	central	2.87 (4.30)	2.62 (4.15)	3.51 (4.71)	2.48 (3.13)	0.84 (3.25)	1.97 (2.92)
	central–parietal	3.32 (3.99)	3.51 (3.79)	3.93 (4.21)	3.30 (2.76)	1.72 (3.16)	2.73 (2.81)
	parietal	2.92 (3.07)	3.34 (3.25)	3.32 (3.38)	3.68 (3.35)	2.24 (2.63)	3.04 (2.64)

**Table 5:** Average energy of alpha waves in different regions [*M* (*SD*)].

	Area	Depression-Prone Group			Control Group		
		Positive	Neutral	Negative	Positive	Neutral	Negative
300–450 ms	prefrontal	−0.27 (0.21)	−0.30 (0.20)	−0.28 (0.17)	−0.22 (0.16)	−0.21 (0.16)	−0.20 (0.19)
	frontal-central	−0.30 (0.24)	−0.33 (0.22)	−0.30 (0.21)	−0.24 (0.17)	−0.24 (0.19)	−0.23 (0.18)
	central	−0.33 (0.25)	−0.36 (0.23)	−0.34 (0.23)	−0.28 (0.18)	−0.28 (0.20)	−0.26 (0.19)
	central-parietal	−0.35 (0.24)	−0.37 (0.22)	−0.34 (0.23)	−0.29 (0.18)	−0.30 (0.21)	−0.27 (2.20)
	parietal	−0.34 (0.27)	−0.37 (0.24)	−0.32 (0.25)	−0.28 (0.23)	−0.29 (0.21)	−0.27 (0.23)
460–660 ms	prefrontal	−0.27 (0.23)	−0.31 (0.23)	−0.30 (0.22)	−0.20 (0.19)	−0.19 (0.19)	−0.13 (0.25)
	frontal-central	−0.31 (0.27)	−0.34 (0.25)	−0.33 (0.23)	−0.22 (0.21)	−0.23 (0.21)	−0.17 (0.25)
	central	−0.36 (0.27)	−0.39 (0.25)	−0.38 (0.24)	−0.26 (0.22)	−0.28 (0.23)	−0.22 (0.25)
	central-parietal	−0.38 (0.26)	−0.40 (0.26)	−0.38 (0.23)	−0.26 (0.22)	−0.31 (0.24)	−0.24 (0.25)
	parietal	−0.38 (0.28)	−0.39 (0.26)	−0.37 (0.24)	−0.27 (0.24)	−0.31 (0.24)	−0.23 (0.27)

## Data Availability

Dataset available on request from the corresponding authors.

## References

[B1-behavsci-16-00666] Aleksandra M., Neil W. B., Oscar W. M., Prabhavi N. P., Paul B. F. (2023). Alterations in EEG functional connectivity in individuals with depression: A systematic review. Journal of Affective Disorders.

[B2-behavsci-16-00666] Angst J., Merikangas K. (1997). The depressive spectrum: Diagnostic classification and course. Journal of Affective Disorders.

[B3-behavsci-16-00666] Auerbach R. P., Stanton C. H., Proudfit G. H., Pizzagalli D. A. (2015). Self-referential processing in depressed adolescents: A high-density event-related potential study. Journal of Abnormal Psychology.

[B4-behavsci-16-00666] Besche-Richard C., Passerieux C., Hardy-Baylé M. C. (2002). Lexical decision tasks in depressive patients: Semantic priming before and after clinical improvement. European Psychiatry.

[B5-behavsci-16-00666] Bourke C., Douglas K., Porter R. (2010). Processing of facial emotion expression in major depression: A review. Australian and New Zealand Journal of Psychiatry.

[B6-behavsci-16-00666] Cai Q., Brysbaert M. (2010). SUBTLEX−CH: Chinese word and character frequencies based on film subtitle. PLoS ONE.

[B7-behavsci-16-00666] Choi S. W., Chi S. E., Chung S. Y., Kim J. W., Ahn C. Y., Kim H. T. (2010). Is alpha wave neurofeedback effective with randomized clinical trials in depression? A pilot study. Neuropsychobiology.

[B8-behavsci-16-00666] Cuijpers P., Pineda B. S., Ng M. Y., Weisz J. R., Muñoz R. F., Gentili C., Quero S., Karyotaki E. (2021). A meta-analytic review: Psychological treatment of subthreshold depression in children and adolescents. Journal of the American Academy of Child and Adolescent Psychiatry.

[B9-behavsci-16-00666] Disner S. G., Beevers C. G., Haigh E. A., Beck A. T. (2011). Neural mechanisms of the cognitive model of depression. Nature Reviews Neuroscience.

[B10-behavsci-16-00666] Duque A., Vázquez C. (2015). Double attention bias for positive and negative emotional faces in clinical depression: Evidence from an eye-tracking study. Journal of Behavior Therapy and Experimental Psychiatry.

[B11-behavsci-16-00666] Elliott R., Zahn R., Deakin J. F. W., Anderson I. M. (2011). Affective cognition and its disruption in mood disorders. Neuropsychopharmacology.

[B12-behavsci-16-00666] Ellis A. J., Beevers C. G., Wells T. T. (2011). Attention allocation and incidental recognition of emotional information in dysphoria. Cognitive Therapy and Research.

[B13-behavsci-16-00666] Faul F., Erdfelder E., Buchner A., Lang A. G. (2009). Statistical power analyses using G* Power 3.1: Tests for correlation and regression analyses. Behavior Research Methods.

[B14-behavsci-16-00666] Fischler I., Bradley M. (2006). Event-related potential studies of language and emotion: Words, phrases, and task effects. Progress in Brain Research.

[B15-behavsci-16-00666] Frühholz S., Jellinghaus A., Herrmann M. (2011). Time course of implicit processing and explicit processing of emotional faces and emotional words. Biological Psychology.

[B16-behavsci-16-00666] Herbert C., Kissler J., Junghöfer M., Peyk P., Rockstroh B. (2006). Processing of emotional adjectives: Evidence from startle EMG and ERPs. Psychophysiology.

[B17-behavsci-16-00666] Jensen O., Mazaheri A. (2010). Shaping functional architecture by oscillatory alpha activity: Gating by inhibition. Frontiers in Human Neuroscience.

[B18-behavsci-16-00666] Joormann J. (2010). Cognitive inhibition and emotion regulation in depression. Current Directions in Psychological Science.

[B19-behavsci-16-00666] Joormann J., Tanovic E. (2015). Cognitive vulnerability to depression: Examining cognitive control and emotion regulation. Current Opinion in Psychology.

[B20-behavsci-16-00666] Kanske P., Kotz S. A. (2007). Concreteness in emotional words: ERP evidence from a hemifield study. Brain Research.

[B21-behavsci-16-00666] Kiang M., Farzan F., Blumberger D. M., Kutas M., McKinnon M. C., Kansal V., Rajji T. K., Daskalakis Z. J. (2017). Abnormal self-schema in semantic memory in major depressive disorder: Evidence from event-related brain potentials. Biological Psychology.

[B22-behavsci-16-00666] Klawohn J., Bruchnak A., Burani K., Meyer A., Lazarov A., Bar-Haim Y., Hajcak G. (2020). Aberrant attentional bias to sad faces in depression and the role of stressful life events: Evidence from an eye-tracking paradigm. Behaviour Research and Therapy.

[B23-behavsci-16-00666] Koster E. H., De Lissnyder E., Derakshan N., De Raedt R. (2011). Understanding depressive rumination from a cognitive science perspective: The impaired disengagement hypothesis. Clinical Psychology Review.

[B24-behavsci-16-00666] Kovacs M., Beck A. T. (1978). Maladaptive cognitive structures in depression. The American Journal of Psychiatry.

[B25-behavsci-16-00666] LeMoult J., Gotlib I. H. (2019). Depression: A cognitive perspective. Clinical Psychology Review.

[B26-behavsci-16-00666] Lewis K. L., Taubitz L. E., Duke M. W., Steuer E. L., Larson C. L. (2015). State rumination enhances elaborative processing of negative material as evidenced by the late positive potential. Emotion.

[B27-behavsci-16-00666] Lyons M., Aksayli N. D., Brewer G. (2018). Mental distress and language use: Linguistic analysis of discussion forum posts. Computers in Human Behavior.

[B28-behavsci-16-00666] Peckham A. D., McHugh R. K., Otto M. W. (2010). A meta-analysis of the magnitude of biased attention in depression. Depression and Anxiety.

[B29-behavsci-16-00666] Schupp H. T., Cuthbert B. N., Bradley M. M., Cacioppo J. T., Ito T., Lang P. J. (2000). Affective picture processing: The late positive potential is modulated by motivational relevance. Psychophysiology.

[B30-behavsci-16-00666] Shane M. S., Peterson J. B. (2007). An evaluation of early and late stage attentional processing of positive and negative information in dysphoria. Cognition and Emotion.

[B31-behavsci-16-00666] Wang X., Shangguan C., Lu J. (2019). Time course of emotion effects during emotion-label and emotion-laden word processing. Neuroscience Letters.

[B32-behavsci-16-00666] Xie X. M., Sha S., Cai H., Liu X., Jiang I., Zhang L., Wang G. (2024). Resting-state alpha activity in the frontal and occipital lobes and assessment of cognitive impairment in depression patients. Psychology Research and Behavior Management.

[B33-behavsci-16-00666] Zhang J., Wu C., Yuan Z., Meng Y. (2019). Differentiating emotion-label words and emotion-laden words in emotion conflict: An ERP study. Experimental Brain Research.

